# The implication of DNA bending energy for nucleosome positioning and sliding

**DOI:** 10.1038/s41598-018-27247-x

**Published:** 2018-06-11

**Authors:** Guoqing Liu, Yongqiang Xing, Hongyu Zhao, Lu Cai, Jianying Wang

**Affiliations:** 1 School of Life Science and Technology, Inner Mongolia University of Science and Technology, Baotou, 014010 China; 2 Inner Mongolia Key Laboratory for Functional Genome Bioinformatics, Inner Mongolia University of Science and Technology, Baotou, 014010 China

## Abstract

Nucleosome not only directly affects cellular processes, such as DNA replication, recombination, and transcription, but also severs as a fundamentally important target of epigenetic modifications. Our previous study indicated that the bending property of DNA is important in nucleosome formation, particularly in predicting the dyad positions of nucleosomes on a DNA segment. Here, we investigated the role of bending energy in nucleosome positioning and sliding in depth to decipher sequence-directed mechanism. The results show that bending energy is a good physical index to predict the free energy in the process of nucleosome reconstitution *in vitro*. Our data also imply that there are at least 20% of the nucleosomes in budding yeast do not adopt canonical positioning, in which underlying sequences wrapped around histones are structurally symmetric. We also revealed distinct patterns of bending energy profile for distinctly organized chromatin structures, such as well-positioned nucleosomes, fuzzy nucleosomes, and linker regions and discussed nucleosome sliding in terms of bending energy. We proposed that the stability of a nucleosome is positively correlated with the strength of the bending anisotropy of DNA segment, and both accessibility and directionality of nucleosome sliding is likely to be modulated by diverse patterns of DNA bending energy profile.

## Introduction

By folding and wrapping with the aid of proteins, eukaryotic genomes are packaged into high-order chromatin structure. Genome packaging is of great interest not because it condenses long DNA molecules to accommodate to the confined space of nucleus, but because chromatin architecture, which is the product of the packaging, regulates or gets involved in many fundamentally important molecular processes, such as DNA replication, transcription, and recombination. Genome is packaged under the control of packaging code in the cell, which indirectly ensures that the packaged chromatin has to be accessible for DNA-involved processes at some regions. Obviously, the current way of the genome packaging as well as the packaging code is the product of the natural selection. The mechanism of the packaging has been a intensively studied issue in the past thirty years^1–5^, but still remains elusive in many aspects, even at the level of nucleosome positioning. Nucleosome, the repeating unit of chromatin, is the first-step product of the packaging and act as a fine-scale regulator of various molecular processes. For example, nucleosome depletion at upstream region of a gene is known to facilitate transcription by increasing chromatin accessibility for transcription factor binding^2^. Numerous factors have been shown to affect nucleosome positioning. Intrinsic DNA properties^6–10^ known to affect nucleosome positioning include 10-bp periodical occurrence of dinucleotides (AA/TT/TA/AT or CC/GG/CG/GC), nucleosome positioning motifs, poly(A) tract, etc. Some non-DNA factors^11–21^ can also affect nucleosome positions. For example, the chromatin remodeling complexes can displace a nucleosome in a ATP-dependent manner^18–21^, while chemical modifications to histones in a nucleosome may recruit other molecules to alter chromatin structure indirectly^17^. The binding competency of genomic regions with histones and other proteins, such as transcription factors and RNA polymerases, can also affect nucleosome formation^13,14^. Besides, nucleosome organization is subjected to both the concentration of histones in a cell and the kinetics of nucleosome formation^4,22,23^. What we focus on in this study is physical aspects of the sequence preference. Most of the positioning signals encoded in DNA sequence can be explained from the perspective of physics. For example, the 10-bp periodicity and positioning motifs in nucleosomal DNA facilitate the wrapping of the sequence around a histone octamer by increasing sequence curvature^5,24^. Among the existing models^25–46^ designed to predict nucleosome positions, physical models^32–46^ are preferable in terms of providing a much deeper and intuitive insight into the nucleosome positioning mechanism. For example, a physical model in which both DNA elastic energy and histone-DNA interaction term used to penalize the deviation of nucleosomal DNA from ideal superhelix were considered successfully predicted *in vitro* nucleosome positions and free energies^40^. In another biophysical model, in addition to sequence-dependent harmonic energy, interactions between histones and nucleosomal DNA at their binding sites were considered^41^. Using the model, the authors predicted positions of several nucleosomes with a high accuracy and investigated the nucleosome sliding supposed to take place through twist defect diffusion. In our previous study, we presented a deformation energy model and successfully applied it to the prediction of nucleosome dyad positions and occupancy^46^. Particularly, bending energy performed very well in the dyad position prediction^46^. Although a number of energetics models were proposed to predict nucleosome-forming ability of DNA sequences^32–46^, to our best knowledge, none of them attempted to discuss the link between DNA deformation energy profile and chromatin remodeling. In this study, we characterized distinctly-organized chromatin structure in terms of DNA bending energy, and explored bending energy-related properties in nucleosome sliding. We also predicted nucleosome formation free energy using our bending energy model. Our results highlight the roles of bending energy in rotational positioning of nucleosomes, nucleosome reconstitution *in vitro* and nucleosome sliding.

## Materials and Methods

### Materials

A well-established nucleosome positioning sequence, 601 sequence, was taken from van der Heijden *et al*.^31^. A 601 sequence-based DNA segment used in a nucleosome sliding experiment was taken from Blosser *et al*.^47^. A unique nucleosome map with base-pair resolution for the yeast genome (sacCer2 version) was taken from Brogaard *et al*.^48^. From the map, we selected top 5000 strong nucleosomes (ratio > 3.604), 5000 medium nucleosomes (1.551 < ratio < 1.793) and bottom 5000 weak nucleosomes (ratio < 0.553) according to NCP/noise ratio. The 5000 medium nucleosomes have ratio values around the mean of all ratio values. NCP here represents nucleosome center positioning score, which measures the nucleosome positioning signal strength and the noise represents the level of background non-specific cleavage of DNA backbone^48^. The larger the ratio is, the higher the strength of placing a nucleosome center at the position. Genomic coordinates of well-positioned nucleosomes, fuzzy nucleosomes and linker regions (S288C) were taken from Lee *et al*.^49^. The genomes of budding yeast (sacCer2 and S288C) were downloaded respectively from UCSC (http://genome.ucsc.edu/) and Saccharomyces Genome Database (https://www.yeastgenome.org/). The sequences used in nucleosome re-constitution experiments *in vitro* and corresponding free energy data were taken from references^50–52^.

Transcription start sites (TSS) for protein-coding genes in budding yeast were obtained from high-resolution transcription map^53^. We selected 4,197 poly(A) RNA hybridization-based transcription segments, which overlap >50% of protein-coding regions of experimentally verified genes located on the same strand. The end sites of the transcription segments, which were located at the 5′ side of the genes, were defined as TSS. Note that in order to analyze the nucleosome map^48^ (sacCer2-based) at the aforementioned TSS (sacCer1-based), the genomic coordinates for the 4,197 transcription segments were converted from sacCer1 to sacCer2 version using LiftOver at UCSC (http://genome.ucsc.edu/). Numerous studies^4,12,48^ shows that most NFR in yeast are roughly located at the position −175 to −25 relative to TSS. Accordingly, the nucleosome-free region (NFR) at 5′ end of protein-coding genes in this study were defined as the region located from −175 to −25, relative to the TSS. The −1 nucleosomes and +1 nucleosomes surrounding the NFR were defined as those whose central genomic positions are located, respectively, from −325 to −175 and −25 to 125. We also analyzed precisely identified −1/+1 nucleosomes, whose genomic coordinates (sacCer3-based) and corresponding version of the genome (sacCer3 version) were derived, respectively, from Table S1 of the literature^54^ and UCSC.

### Bending energy calculation

A nucleosome consists of a histone octamer and a DNA segment of 147 bp that is sharply bent and tightly wrapped ~1.7 times around the histone octamer in a left-handed superhelix. Two 9-bp ends of nucleosomal DNA have little contribution to its curvature^24,46^ and hence a 129-bp window is used in bending energy calculation. The bending energy is formulated as

1 $${E}_{b}(i)=\sum _{i=1}^{128}\,[\frac{1}{2}{k}_{\rho }(i){[\rho (i)-{\rho }_{0}(i)]}^{2}+\frac{1}{2}{k}_{\tau }(i){[\tau (i)-{\tau }_{0}(i)]}^{2}]$$

where *ρ*(*i*) and *τ*(*i*) are, respectively, the predicted roll and tilt angles at dinucleotide step *i* in a 129-bp DNA segment assumed to be subject to a constraint of curvature of 579°, which is the same as that for the central 129-bp part of the ideal superhelix that best fits the core DNA in the nucleosome core particle^24^; *ρ*_0_(*i*) and *τ*_0_(*i*) are equilibrium values of roll and tilt respectively for the dinucleotide at step *i*; *k*_*ρ*_(*i*) and *k*_*τ*_(*i*) are the dinucleotide-dependent force constants. Here, roll and tilt are two of the six degrees of freedom used to describe the geometry of DNA double helix according to Cambridge Convention^55^. The sequence-dependent force constants and equilibrium values of roll, tilt and twist used in this study were all taken from Liu *et al*.^46^, which were estimated by using the structures of protein-DNA complexes.

As previously done^46^, *ρ*(*i*) and $\tau (i)$ in Eq. 1 were estimated by using the structure constraint (Eq. 2) derived from a nucleosome crystal structure and the relation (Eq. 3) between deformations in roll and tilt and bending force supposed to be uniformly distributed along the DNA sequence analyzed. Cumulative helical twist at step *i*, ${{\rm{\Omega }}}_{i}$, was calculated by adding up the equilibrium twists of the dinucleotide steps counted from the central base-pair of the sequence.

2 $${579}^{\circ }=\sum _{i=1}^{128}[\rho (i)\cos \,{{\rm{\Omega }}}_{i}+\tau (i){\sin {\rm{\Omega }}}_{i}]$$

3 $$\{\begin{array}{c}\rho (i)-{\rho }_{0}(i)={F}_{b}\,\cos \,{{\rm{\Omega }}}_{i}/{k}_{\rho }(i)\\ \tau (i)-{\tau }_{0}(i)={F}_{{\rm{b}}}\,\sin \,{{\rm{\Omega }}}_{i}/{k}_{\tau }(i)\end{array}$$

Bending energy of any sequence segment, 129 bp in size, is computed by combining the aforementioned three equations. The unit of force constants used in our model is k_b_T/degree^2^, and hence the unit of bending energy calculated with Eq. 1 is k_b_T, where k_b_ is Boltzmann constant and T is effective temperature. After dividing the bending energy by 128, the number of base-pair steps of the sequence segment, we obtain average bending energy per base-pair step, whose unit is k_b_T/bps where bps denotes base-pair step.

**Figure 1:**
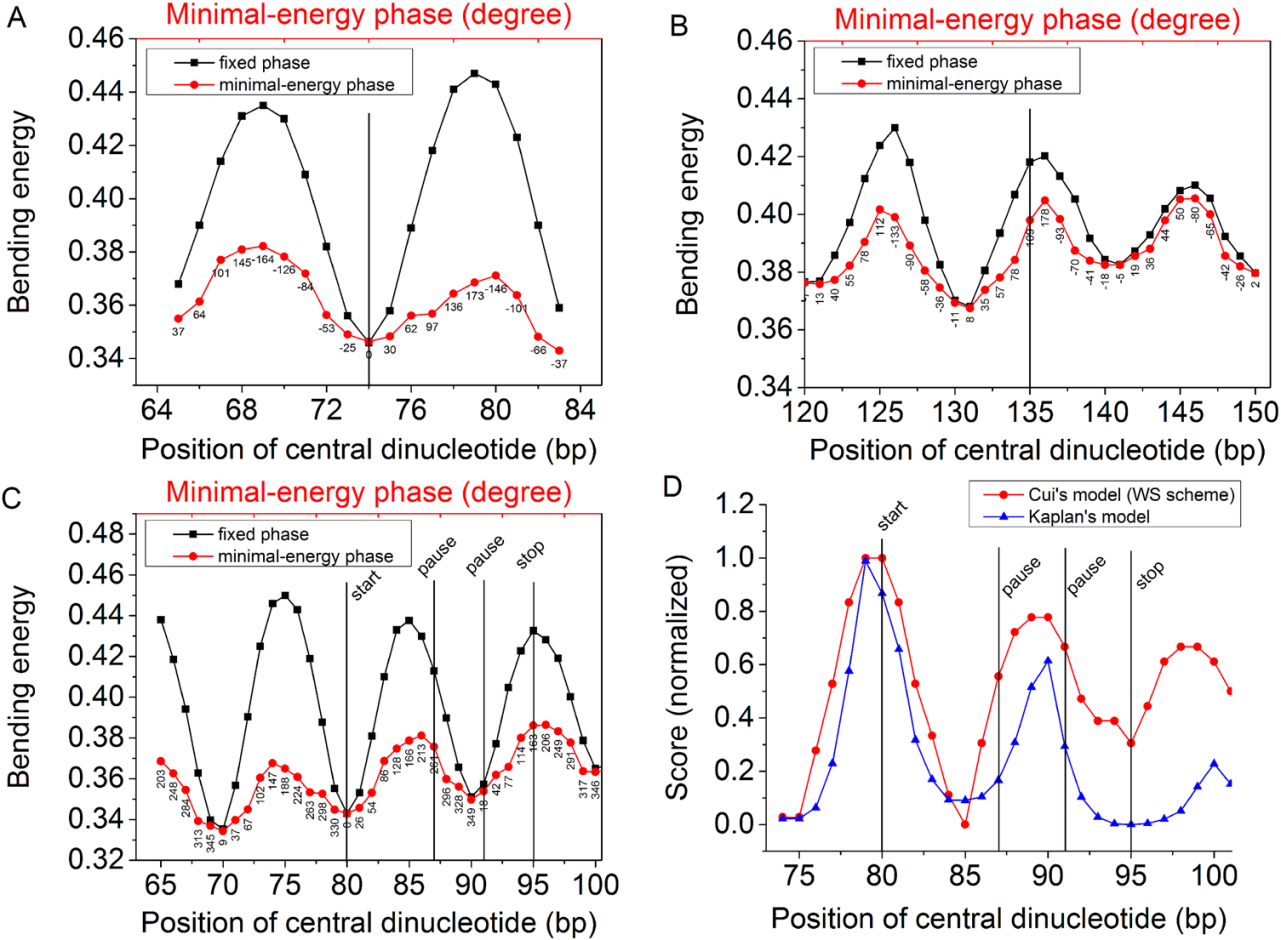
Bending energy profiles for sequences. The units of bending energies reported throughout the results are k_b_T/bps. (**A**) Bending energies for 601 sequence were calculated using “fixed model” and “minimal-energy model”. The phases of the central dinucleotide of 129-bp window corresponding to minimal energies in “minimal-energy” model was indicated under the plot symbols. (**B**) Bending energies for 5S oocyte sequence. (**C**) Bending energies for a 601 sequence-based DNA segment, which was used in a nucleosome sliding experiment *in vitro*^47^. The “start”, “pause” and “stop” in the figure represent the positions at which the nucleosome was initially positioned, transiently paused, and finally reached, respectively. (**D**) For the 601 sequence-based DNA segment, score of a nucleosome center being at the positions along the DNA sequence were calculated using Cui’s model^55^ and Kaplan’s model^4^. The original scores were normalized to the range of 0–1 for the convenience of display.

Bending energy is inversely correlated with nucleosome-forming ability of a DNA segment. In other words, the lower the bending energy of a sequence is, the easier the sequence is to form a nucleosome. The equation for bending energy calculation where the central dinucleotide of analyzed DNA segment was assigned a fixed phase of zero was designed to estimate the bending energy of the DNA segment which bends toward the major groove at its central dinucleotide (see Liu *et al*.^46^ for details). To assess the maximal bendability that a DNA segment can have, a minor modification was made: we first calculated bending energies corresponding to every bending direction toward which the central dinucleotide bends, by altering the phase of the central dinucleotide from −180 degrees to 180 degrees, and then picked out the minimal energy to assess the maximal bendability. In a word, the present study is mainly an application of our previous model and only one methodological extension of the model is the measurement of minimal bending energy for a DNA sequence by selecting the smallest one from computed bending energies that correspond to different directions in which the DNA bends. In this study, the previous bending energy model is denoted as “fixed phase” model, and the modified one as “minimal energy phase” model. Throughout the manuscript, “fixed phase” model was used unless stated. To be specific, “minimal energy phase” model was used only in Fig. 1. The bending energy profile difference between the two models was shown taking “601” sequence as an example (Fig. 1A). Although minor difference exists, both models indicated a local energy minimum at its central position, at which the “601” sequence bends toward its major groove.

In our model, twist deformation and DNA stretching that play some roles in nucleosome positioning are not considered. The twist term, however, is coupled with other degrees of freedom used to describe DNA structure, particularly with the roll as illustrated by crystal structure of NCP147. There is a strong correlation between roll and twist^32^. So, the inclusion of roll, which is a major contributor to DNA bending, in our model automatically takes at least a significant part of the twist effect into account. Furthermore, in the calculation of bending energy, we used dinucleotide-dependent cumulative twists that indicate proper phasing of dinucleotides relative to the dyad position of a nucleosome, and the use of the dinucleotide-dependent twists instead of a constant twist for all dinucleotide steps represents the inclusion of twist effect to some extent in our model. In addition, what we focus on in the study is the effect of DNA bending on rotational positioning and sliding of nucleosomes, in which the stretching and shearing of DNA sequence are not as important as bending.

### Distribution of 10-bp periodicity, fluctuation amplitude and minimal value of bending energies along genomic sequences

We first obtained the series of bending energies for each of the genomic regions for well-positioned nucleosomes, fuzzy nucleosomes and linkers using “fixed phase model”, and then carried out following analyses.

Fast Fourier Transform algorithm was employed to detect periodical signals in each series of bending energies^46^ using a sliding window of 50. For N-sized numerical data, its Fourier transform $F(k)$ is given by

4 $$F(k)=\sum _{m=0}^{N-1}E(m){e}^{-2\pi mik/N},\,k=0,1,\cdots ,N-1$$

where N is a positive even number. Periodicity is denoted as $f=N/k$. Practically, it is enough to analyze the Fourier transform with *k* in the range [0, *N*/2], as the amplitude of the Fourier transform, $|F(k)|$, is symmetric with respect to $k=N/2$. To capture an amplitude corresponding to ~10-bp periodicity, we selected the maximal value of the amplitudes in the periodicity range between 9.5 and 11.5 to represent the intensity of ~10-bp periodicity.

The distribution of fluctuation amplitudes of bending energies along the genomic sequence was obtained by consecutively computing the absolute of the difference between each pair of maximum and minimum within a sliding window of 12 along the series of bending energies. The window size, 12, was selected to capture the local maximum and minimum within each oscillating period (~10 bp) of the calculated bending energies, which corresponds to the bending anisotropy of DNA. The distribution of energy minima was obtained similarly using a sliding window of 12 along the series of bending energies by selecting minimum within the window. Average distribution profiles of aforementioned three indexes for each kind of genomic sequences, such as well-positioned nucleosomes, fuzzy nucleosomes and linkers, were obtained respectively by averaging over the same kind of genomic sequences.

### Definition of canonical positioning/alternative positioning

Based on the bending energies derived from the fixed-phase model, we defined two nucleosome positioning modes: canonical positioning and alternative positioning. A nucleosome is considered to adopt canonical positioning mode if its nucleosomal DNA sequence has a local energy minimum for its central subsequence of 129 bp, and if no local energy minimum is detected at the central position, it is called alternative positioning. A maximal uncertainty of 2 bp is allowed for the “central position”. Besides, in order to exclude biologically meaningless local energy minima which are the result of random fluctuation of bending energies, the “local energy minimum” here is defined as the lowest and central value among five consecutive bending energies, which monotonically decrease from its both sides.

### Spearman rank correlation

Spearman rank correlation analysis was performed using SPSS 11.5 to test the correlation between two *n-*sized variables, and correlation coefficients (*R*) and *P* values were reported. Whether the observed Spearman rank correlation coefficient is significantly different from zero was tested using ${t}={R}\sqrt{\frac{{n}-{\rm{2}}}{({1}-{{R}}^{2})}}$, which is distributed approximately as Student’s *t* distribution with *n* − 2 degrees of freedom under the null hypothesis stating that there is no relationship between the two variables.

## Results and Discussion

### Prediction of nucleosome dyad positions

In our previous study^46^, we successfully predicted dyad positions for 19 out of 20 nucleosomes that were re-constituted *in vitro*. To test if the model established on minimal energy phase frame could give any implication on the positioning mechanism of one wrongly predicted nucleosome whose dyad position is at 135 bp on 5S oocyte sequence, we presented the bending energy profile for the nucleosome. Unfortunately, no expected local energy minimum was detected at the position 135 (Fig. 1B).

### Bending energy profile in nucleosome sliding

What we are interested in this study is “can we infer some information from bending energy profile of sequence if nucleosome sliding is influenced by DNA sequence?”. Motivated by this question, we analyzed bending energy profile for a 601 sequence-based DNA segment, which was used in a nucleosome sliding experiment *in vitro*^47^. A nucleosome was assembled on the sequence, and then remodeled under the ATP-dependent action of a remodeler protein, ACF. The nucleosome was initially positioned at a position denoted as “start” in Fig. 1C, and then moved and transiently paused twice before it reached its final state (denoted as “stop” in Fig. 1C) on the sequence. We expect possible bending energy minima at the positions including start, pause and stop positions, which indicate relatively stable states of binding between histone octamer and underlying sequence. We really observed local energy minima at the initially positioned position and one intermediately paused position as expected. However, the bending energies at the other intermediately paused position and finally reached position do not tend to adopt low bending energy, but instead show relatively high local energies. In addition, we tried two other state of the art models^6,56^ for predicting nucleosome center positions and found that the models also failed to predict a peak of positioning score at the “stop” position (Fig. 1D). These results implicate that nucleosomes, particularly *in vivo*, do not necessarily have a representative feature of minimal energy. One possible explanation for this is the energy barriers associated with the DNA-histone interaction, which might occur in the process of nucleosome sliding and block the access of the nucleosome to a minimal energy state. Moreover, for *in vivo* system, some other factors such as the dynamics of nucleosomes may also account for the observed results. Nucleosome maps obtained in different studies show a certain extent of discrepancy, which can be caused by several kinds of noise, such as different experimental conditions, sequencing data processing, cell cycle phase, gene transcription rate, nucleosome dynamics and sample variations produced by differences in the growth media^57^. Flores *et al*.^57^ proposed that nucleosomes in most cases move along the one-dimensional DNA fiber and are mainly positioned at specific places in response to strong nucleosome depletion signals, such as intrinsic properties of DNA, the competition of histones with DNA-binding proteins and chromatin-remodelers. This means nucleosome is a highly dynamic structure in the cell and the noise mentioned above is likely to impede at least a minor proportion of nucleosomes from adopting minimal energy states. For example, if a transcription factor bound to genomic DNA impedes the sliding of a nucleosome before it reaches the state having local energy minimum, the nucleosome may position for a long or short time at a place where there is no local energy minimum. We therefore do not believe that nucleosomes in such a dynamic *in vivo* system are all positioned at genomic sites having local minima of bending energy.

**Figure 2:**
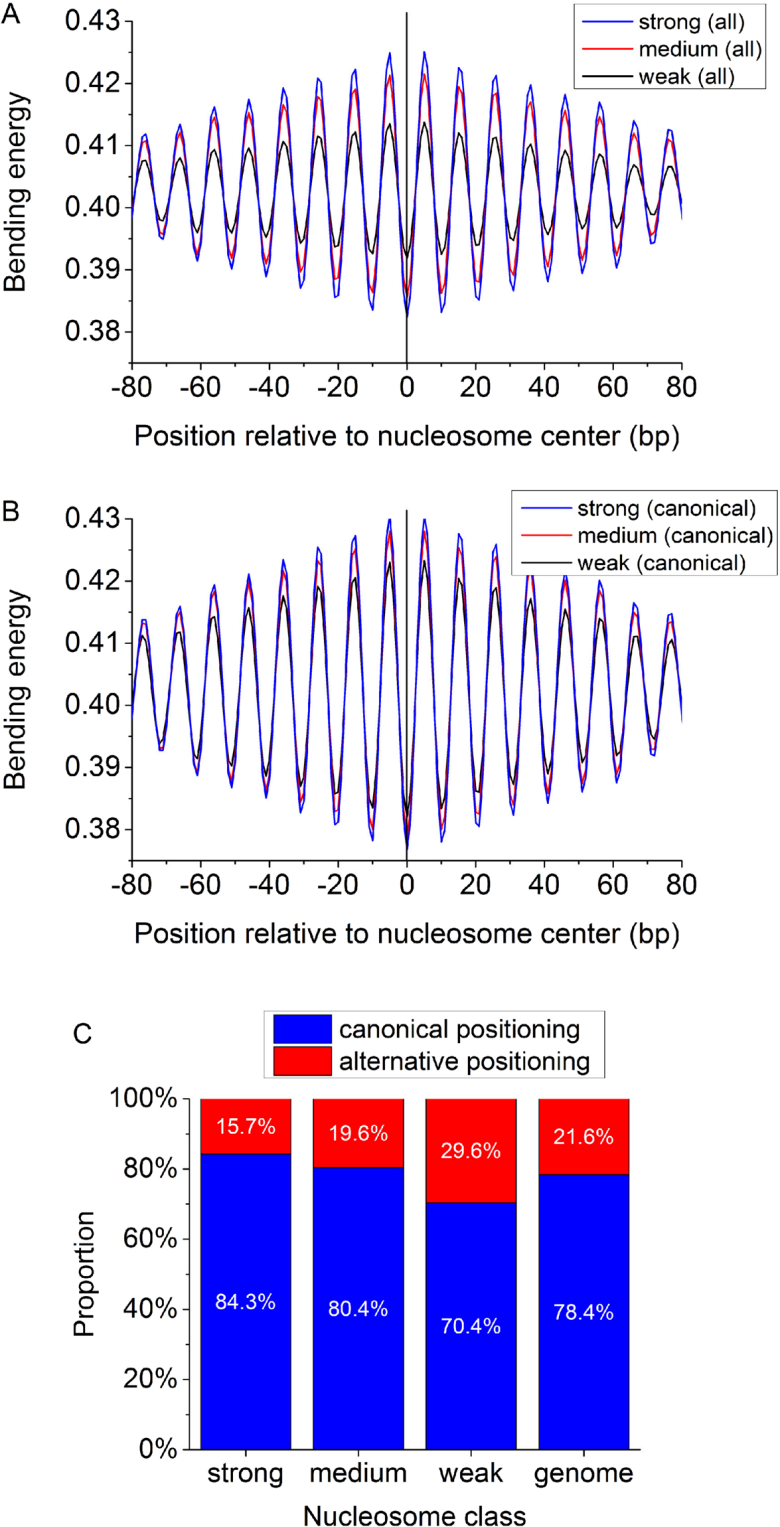
(**A**) Average bending energy profile for nucleosome positioning sequences in the nucleosome map of Brogaard *et al*.^48^. The nucleosome positioning sequences were divided into three categories according to the intensity of nucleosome positioning signal (see “Materials” for details). (**B**) The same as (**A**), but alternative positioning nucleosomes were not included in the analysis. (**C**) The proportion of alternative positioning nucleosomes increases as the nucleosome center positioning signal weakens from strong, medium to weak. “canonical positioning” represents a nucleosome positioning mode in which the nucleosomal sequence has a local bending energy minimum at its center. In contrast, “alternative positioning” does not have a local bending energy minimum at the center of the sequence.

Given that not all nucleosomes can be characterized by a local bending energy minimum, it is interesting to see what proportion of a genome is occupied by such non-canonical nucleosomes without local energy minimum. It is an important question because the extent of the prevalence of this non-canonical positioning pattern can not only help us understand the chromatin structure organization and structure-mediated processes, but also directly affect the accuracy of various sequence-dependent models designed to predict nucleosome positions. It is generally accepted that strong nucleosomal DNA sequences have strong periodical signals^6,11^ and the periodicity encoded in DNA sequences for nucleosome positioning has a close relationship with its bending energy. Dividing 67,543 nucleosomes from the base-pair resolution unique map for yeast^48^ into three categories according to nucleosome center positioning score/noise ratio, we found that as the positioning signal in sequences becomes stronger, the amplitude of the bending energy profile becomes greater (Fig. 2A). There are three possible reasons for the reduced amplitude for the weak nucleosomes. Firstly, the amplitudes of bending energy profiles for individual sequences tend to decrease as the positioning signals in sequences become weaker. Secondly, the bending energy of weak nucleosomal sequences does not show as regular appearance of 10–11 bp periodical oscillation as strong nucleosomes. Thirdly, weak nucleosomes do not have consistently low bending energy at their aligned sites (nucleosome centers), which can result in the averaged weak amplitude profile for weak nucleosomes even if they have equivalent extend of 10-bp periodicity as strong nucleosomes. We tested if the three categories of nucleosomes differ in the proportion of local minimum of bending energy at the aligned sites. Here, a nucleosome positioning mode is called “canonical positioning” if corresponding nucleosomal sequence has a local energy minimum at its center, otherwise called “alternative positioning”. As shown in Fig. 2C, as nucleosome center positioning signal weakens, the proportion of alternative positioning nucleosomes increases from 15.7% to 29.6%, indicating the appearance of local energy minimum at nucleosome centers is not an exclusive unique mode of nucleosome positioning. In yeast, at genome-wide level, about 21% nucleosomes adopt such kind of alternative positioning. Accordingly, we propose that people should be cautious in using a framework which can only predict canonical positioning of nucleosomes and explaining the corresponding results. Moreover, we carried out a similar analysis excluding the alternatively positioned nucleosomes, and reproduced a similar pattern (Fig. 2B) as in Fig. 2A, suggesting that: (1) the difference in bending energy between three categories of nucleosomes cannot be ascribed solely to the alternatively positioned nucleosomes; (2) bending energy is an indicator of nucleosome positioning ability of DNA sequence even when the effect of alternative positioning was removed.

**Figure 3:**
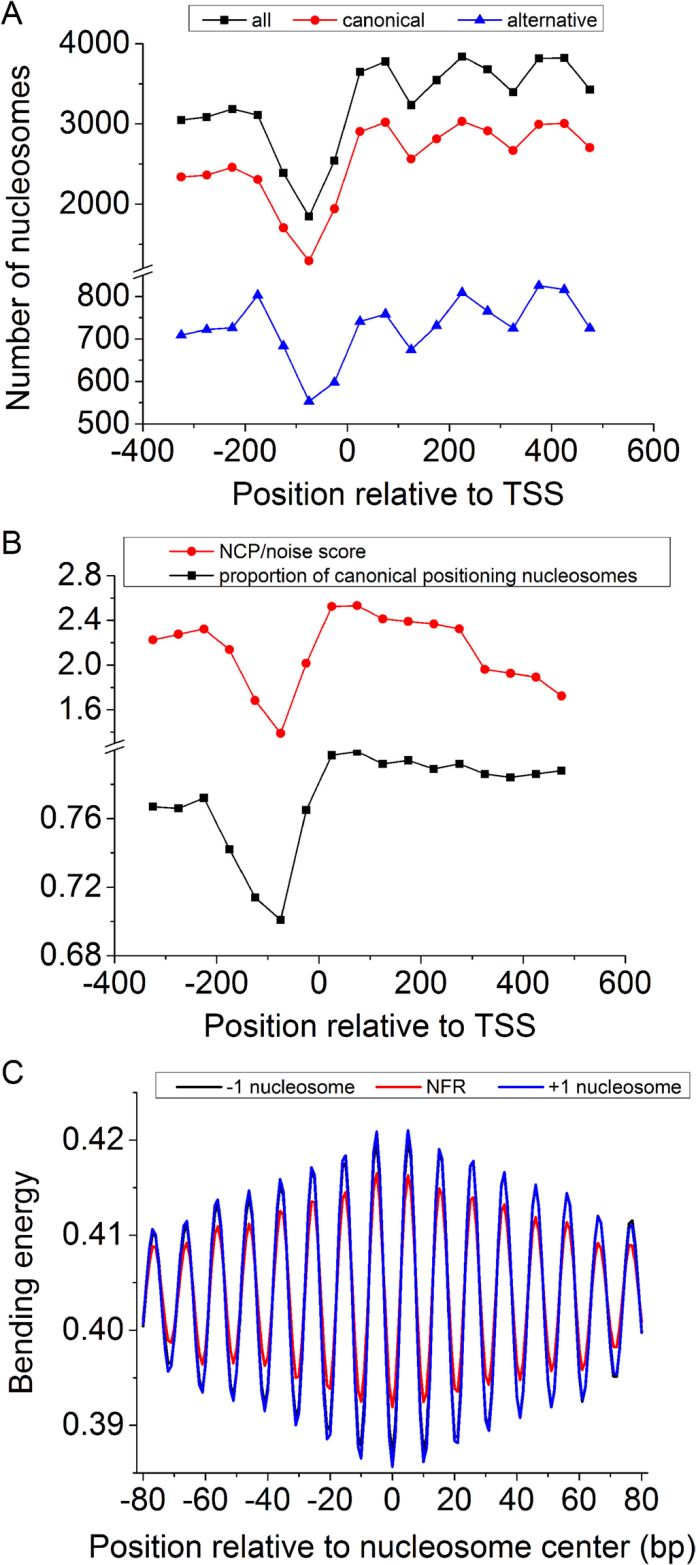
Distribution of nucleosomes around transcription start sites (TSS). The unique nucleosome map^48^ and a total of 4,197 TSS for protein-coding genes^53^ were analyzed here (see Materials for details). (**A**) Both canonical and alternative nucleosomes show similar distribution patterns around TSS. They are depleted at the upstream of the TSS (from position −125 to −25), and enriched in gene body regions. A window of 150 bp with a moving step of 50 bp was used in nucleosome counting. If the center of a nucleosome is located in the predefined window, it is considered to be one hit for that window. (**B**) The proportion of canonical nucleosomes in the predefined window varies around the TSS. The proportion is low for the nucleosome-depleted region and high for surrounding regions where +1 and −1 nucleosomes with high nucleosome center positioning scores (NCP/noise) are located. (**C**) DNA bending energy difference between the nucleosome free regions (NFR, from position −175 to −25), −1 nucleosomes (from −325 to −175), and +1 nucleosomes (from −25 to 125).

In order to see to what extent +1 nucleosomes positioned at the 5′ end of genes are dictated by the bending energy, we analyzed the distribution of nucleosomes around transcription start sites and corresponding DNA bending energies. Our results show that both canonical and alternative nucleosomes are depleted at the upstream of the TSS (from position −125 to −25 in Fig. 3A), and a much higher proportion of +1 and −1 nucleosomes tend to be canonically positioned as compared to the nucleosomes found in the nucleosome-depleted region (Fig. 3B). Furthermore, as compared with the nucleosome free region (NFR), +1 and −1 nucleosomes can be characterized with a lower level of bending energy minima and higher bending anisotropy indicated by the higher amplitude in the bending energy oscillation (Fig. 3C). We also show that precisely identified +1/−1 nucleosomes have slightly enhanced 10-bp oscillation in bending energy than the +1/−1 nucleosomes defined in this study (supplementary Fig. S1).

**Figure 4:**
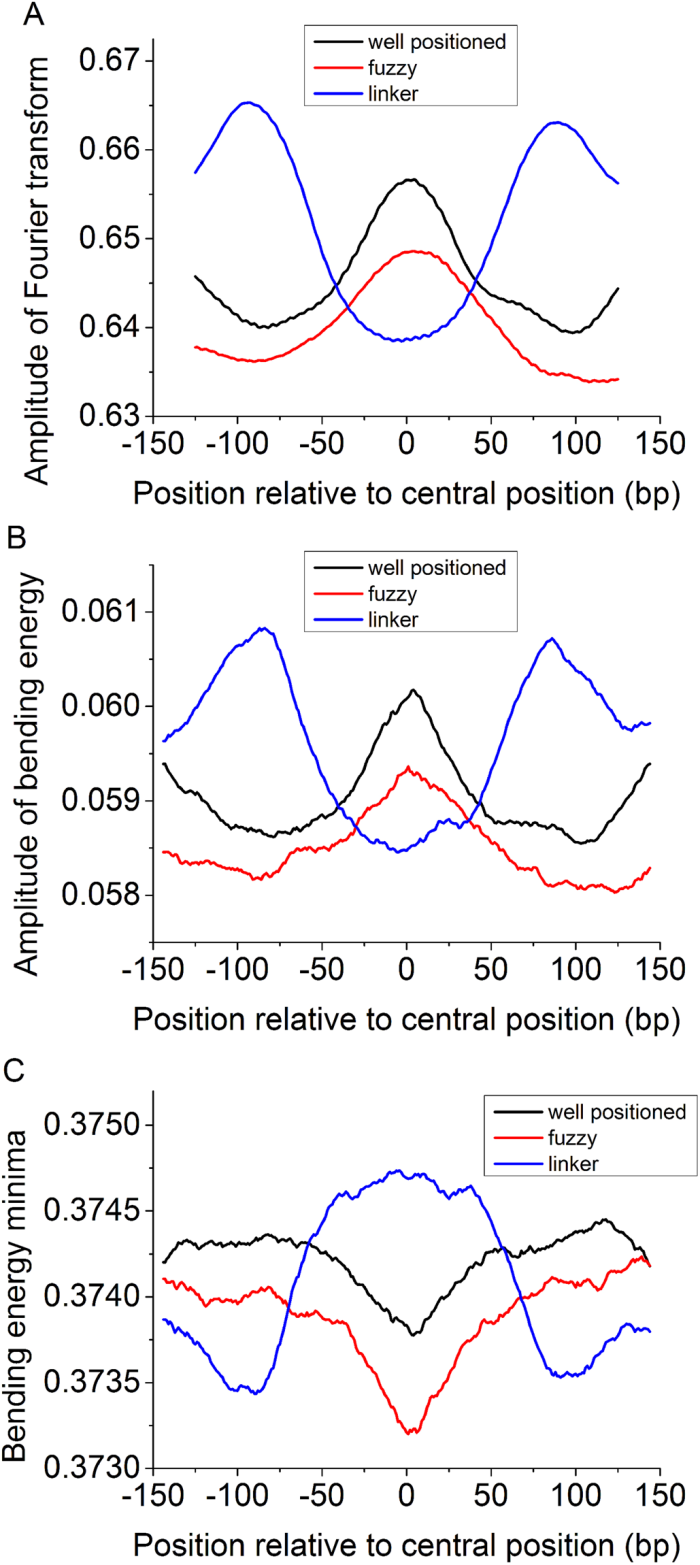
(**A**) Distribution of ~10-bp periodicity amplitudes of the Fourier transform of bending energies for distinctly organized nucleosome regions. (**B**) Distribution of fluctuation amplitudes of bending energies for distinctly organized nucleosome regions. (**C**) Distribution of bending energy minimum for distinctly organized nucleosome regions. See “Materials and Methods” for methodology adopted here.

**Figure 5:**
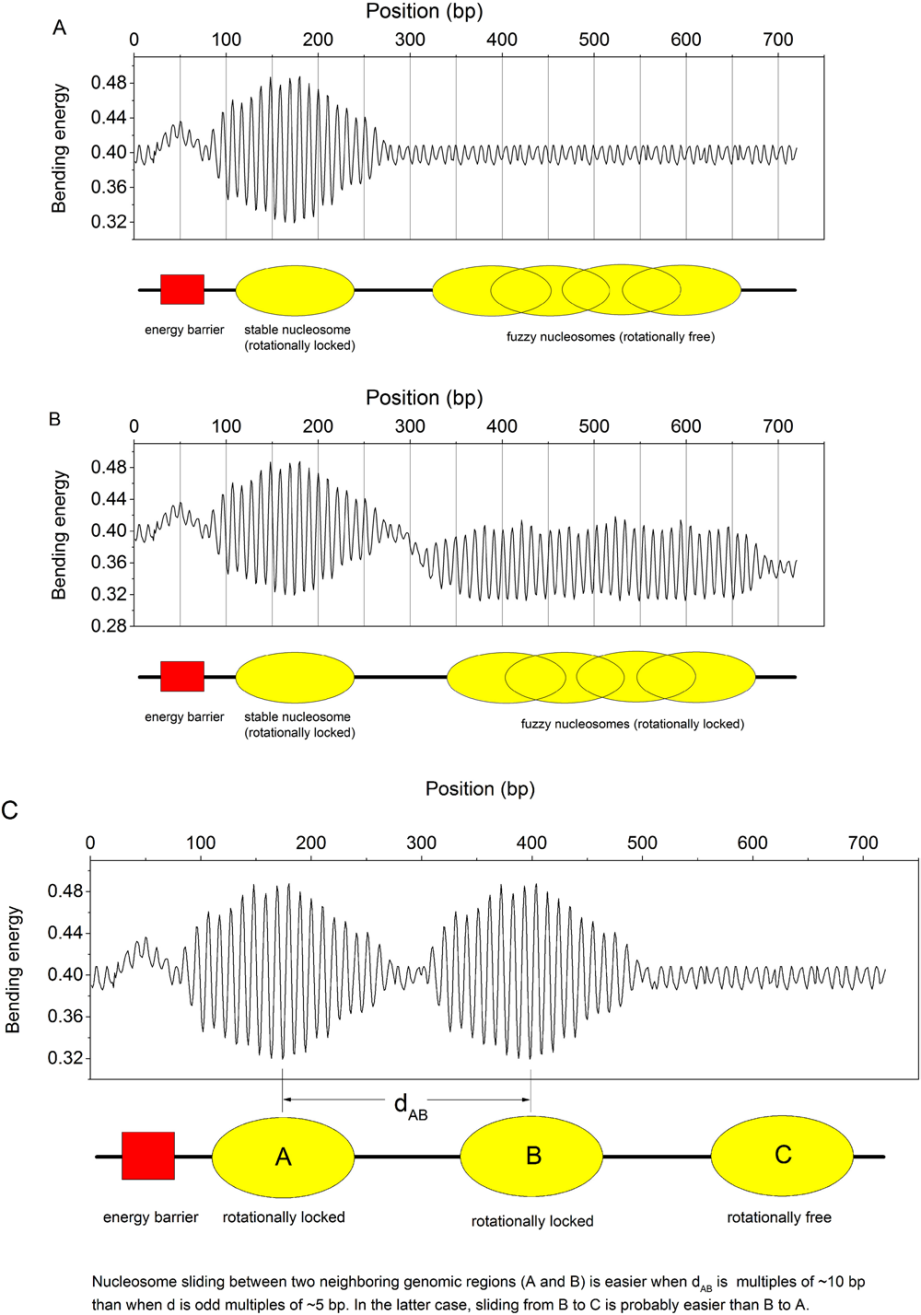
Cartoons of bending energy profiles for diverse nucleosome positioning patterns. The cartoons were drawn only for illustration of nucleosome positioning patterns for different genomic regions and bending energy-dependent model for nucleosome sliding, and therefore the bending energies in the Figure do not represent stringent quantitative predictions of the deformation energy model. (**A**) Pattern of deformation energy profiles (adapted from the reference^18^) for different genomic regions, such as energy barrier regions, well-positioned nucleosomes and fuzzy nucleosomes. (**B**) Pattern of bending energy profiles proposed in this study. (**C**) A bending energy-based model for nucleosome sliding. Nucleosome sliding between two neighboring positioning sites (**A**,**B**) is favored when they have the same rotational setting (their distance is multiples of ~10 bp). In contrast, if they have opposite rotational setting (their distance is odd multiples of ~5 bp) sliding between them is disfavored, and in this case, sliding from (**B**) to a rotationally free site (**C**) is probably easier than sliding to a rotationally opposite site (**A**). In Fig. 5C, the yellow ovals represent three genomic sites, which are likely to accommodate nucleosomes. According to the bending energy profile and our discussion, we know that well-positioned nucleosomes are favored at site (**A** and **B**), while site (**C**) neither represents a well-positioning region, nor a fuzzy nucleosome region. Although the site (**C**) is less favorable for positioning a nucleosome than site (**A** and **B**), it is also represented by an oval to illustrate our nucleosome sliding model.

We are also interested in characterizing linker regions and well-positioned and fuzzy nucleosomes in terms of bending energy. Because 10-bp periodical oscillation of bending energy reflects directly the bending anisotropy of a DNA sequence, which has a close relationship with the rotational positioning of the nucleosome, we analyzed the ~10 periodicity in the bending energies calculated along the sequences by using Fourier transform. As expected, the bending energy of well-positioned nucleosomes exhibits much stronger ~10-bp periodicity than fuzzy nucleosomes and linker sequences (Fig. 4A). The two sides encompassing the linker regions have much elevated ~10-bp periodicity in bending energy (Fig. 4A),which can be ascribed to nucleosome positioning sequences flanking the linker sequences. Consistently, the amplitude of bending energy for well-positioned nucleosomes is the greatest and that for linkers is the smallest (Fig. 4B). It is worth noting that strong periodicity of bending energy does not necessarily mean a low bending energy. As depicted in Fig. 4C, we found an interesting result that the averaged profile of the local bending energy minima is the lowest for fuzzy nucleosomes. According to the aforementioned results, we presented bending energy profiles for diverse nucleosome positioning patterns in Fig. 5B, which differs largely from previously proposed one (Fig. 5A) in the context of fuzzy nucleosomes.

Is it possible to give a further insight into chromatin remodeling from the perspective of sequence bending energy? As previously found, it is difficult for a nucleosome positioned at a genomic region with low bending energy to slide towards regions with high bending energy, particularly when the nucleosome is rotationally locked (Fig. 5C). Besides, given that the rotational setting of the underlying sequence with respect to histone surface can hardly change in the course of nucleosome sliding^18^, we propose here that there is a simple bending energy-based rule directing nucleosome sliding (Fig. 5C): a nucleosome is easy to slide between two neighboring genomic positions if they have the same rotational setting, otherwise, nucleosome sliding is difficult. If a nucleosome slides between two positions with opposite rotational settings, the underlying DNA sequence has to be rotate 180 degrees relative to the double-helix axis to accommodate its rotational setting to histone surface, which can apparently introduce a large energy barrier for nucleosome sliding. In other words, apart from remodeler activity, bending energy profile of DNA sequence may also play a important role in the accessibility and directionality of nucleosome sliding. Note that the model of sequence-dependent nucleosome sliding proposed above is for ATP-dependent sliding of nucleosomes, in which rotational setting of DNA on the histone core remains unaltered as described in loop/bugle propagation model^58^. Among several models proposed for nucleosome sliding^58^, twist defect diffusion model and loop/bugle propagation model are of great interest. Both of the models are supported by some experimental evidence, but a major difference between the models is: the diffusion model for nucleosome sliding along DNA in response to thermal fluctuations predicts an alteration of the rotational phasing of the DNA on the histone core, while the loop propagation model for ATP-dependent sliding of nucleosomes allows DNA to maintain the rotational phasing^58^.

### Nucleosome free energy prediction

**Figure 6:**
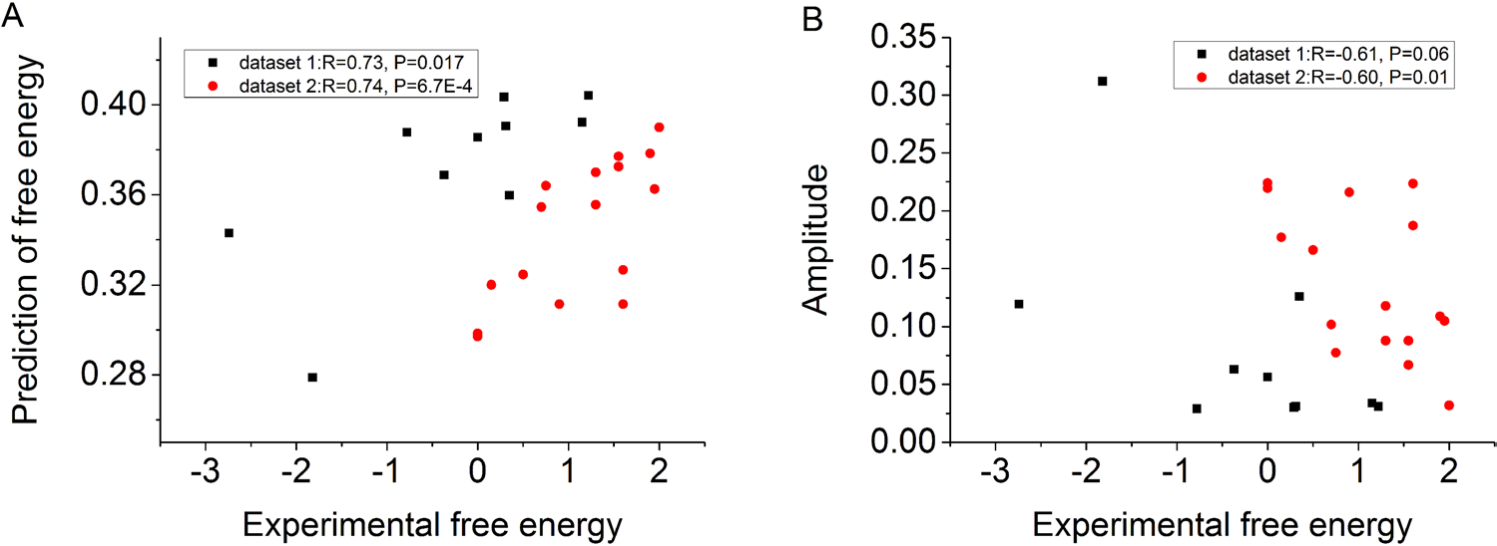
Bending energy is a good indicator of free energy in nucleosome re-constitution experiments *in vitro*. Dataset 1 is a sequence set from reference^50^, and Dataset 2 is a sequence collection from references^51,52^. (**A**) The mean of local minima of bending energies has a significant rank correlation with nucleosome free energies. Spearman correlations coefficients and corresponding *P*-values were reported in the figure (see Method section for the significance test). (**B**) The amplitude of bending energy profile also has a significant rank correlation with nucleosome free energies.

In our previous study, our model performs well in predicting nucleosome dyad positions and occupancy^46^. The model also successfully discriminated nucleosome-enriched regions from nucleosome-depleted regions. However, it is unclear how the model performs in nucleosome free energy prediction. The free energy refers to the change of free energy of the experimental system used to re-constitute nucleosomes *in vitro*. Generally, DNA sequences with low bending energy tend to form nucleosomes and have low free energy in the process of nucleosome assembly *in vitro*. Given the majority of the nucleosomes tend to adopt canonical positioning, the rotational positioning of which can be more clearly captured by our fixed-phase model than minimal-phase model, we developed a free energy prediction model based on the fixed-phase model. A local minimum of calculated bending energy profile represent the ability of the region centering at the minimum to form a nucleosome. In our collected free-energy data, the precise positions of the re-constituted nucleosomes are unknown, raising a question that which bending energy data is suitable for predicting free energy. Because the bending energy minima are all possible positioning sites in nucleosome assembly experiments, we decided to rank nucleosome free energy by the mean of local minima of bending energies. This approach successfully predicted the ranks of the nucleosome free energies (Fig. 6A). We also show that the amplitude of the bending energy profile is significantly anti-correlated with free energy (Fig. 6B), indicating that bending anisotropy of DNA segment can significantly affect nucleosome formation.

## Conclusion

To conclude, we investigated the DNA-encoded signals in chromatin remodeling, characterized distinctly-organized chromatin structure and predicted nucleosome free energies using a DNA bending energy model. Our results show that well-positioned nucleosomes, fuzzy nucleosomes and linker regions have distinct bending energy profiles; approximately, 21% of the nucleosomes in yeast adopted an alternative positioning mode that is different from canonical positioning; DNA bending energy alone can serve as a good indicator of nucleosome forming ability. Our data suggest that the stability of a nucleosome is positively correlated with the strength of the bending anisotropy of DNA segment, and both accessibility and directionality of nucleosome sliding are likely to be modulated by diverse patterns of DNA bending energy profile.

## Electronic supplementary material

Supplementary Figure S1
