# Supplementary material for: The implication of DNA bending energy for nucleosome positioning and sliding
Source: Sci Rep. 2018 Jun 11;8:8853. doi: 10.1038/s41598-018-27247-x (PMC5995830; doi:10.1038/s41598-018-27247-x)
Supplement: Supplementary file 1 — Supplementary Figure S1 [file 41598_2018_27247_MOESM1_ESM.pdf]

Supplementary Information for:
The implication of DNA bending energy for nucleosome positioning and sliding

Guoqing Liu, Yongqiang Xing, Hongyu Zhao, Lu Cai, Jianying Wang

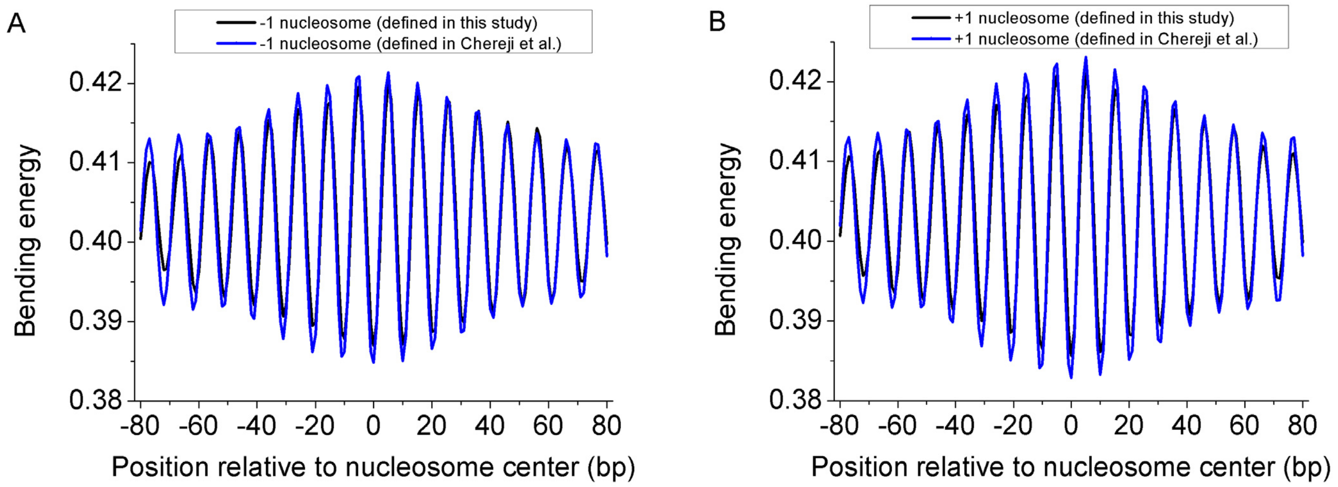

Figure S1. The precisely identified -1/+1 nucleosomes[54] (blue curve) show slightly enhanced 10-bp periodical oscillation of bending energy as compared to the -1/+1 nucleosomes defined in this study (black curve).
